# Fostering Resilience in Pregnancy and Early Childhood During the COVID-19 Pandemic: The HUGS/Abrazos Program Design and Implementation

**DOI:** 10.3389/fpubh.2021.633285

**Published:** 2021-04-28

**Authors:** Meisui Liu, Fernanda Neri Mini, Carlos Torres, Gracia M. Kwete, Alexy Arauz Boudreau, Mary Lyons Hunter, Maria Yolanda Parra, William Lopez, Amy Izen, Sarah N. Price, Meghan E. Perkins, Elsie M. Taveras

**Affiliations:** ^1^Division of General Pediatrics, Department of Pediatrics, Massachusetts General Hospital for Children, Boston, MA, United States; ^2^Harvard Medical School, Boston, MA, United States; ^3^MGH Chelsea HealthCare Center, Chelsea, MA, United States; ^4^MGH Revere HealthCare Center, Revere, MA, United States; ^5^Center for Community Health Improvement, Massachusetts General Hospital, Boston, MA, United States; ^6^Kraft Center for Community Health, Massachusetts General Hospital, Boston, MA, United States; ^7^Department of Nutrition, Harvard T.H. Chan School of Public Health, Boston, MA, United States

**Keywords:** COVID-19, toxic stress, resilience, pregnancy, early childhood, patient navigation

## Abstract

Pregnancy and early childhood pose unique sensitivity to stressors such as economic instability, poor mental health, and social inequities all of which have been magnified by the COVID-19 pandemic. In absence of protective buffers, prolonged exposure to excessive, early adversity can lead to poor health outcomes with significant impact lasting beyond the childhood years. Helping Us Grow Stronger (HUGS/Abrazos) is a community-based program, designed and launched at the time of the COVID-19 surge in the Spring of 2020, that combines emergency relief, patient navigation, and direct behavioral health support to foster family resilience and mitigate the negative impacts of COVID-related toxic stress on pregnant women and families with children under age 6. Through a targeted referral process, community health workers provide resource navigation for social needs, and a social worker provides behavioral health support. The use of innovative tools such as a centralized resource repository, community health workers with specialized knowledge in this age range, and a direct referral system seeks to assist in streamlining communication and ensuring delivery of quality care. We aim to serve over 300 families within the 1st year. The HUGS/Abrazos program aims to fill an important void by providing the necessary tools and interventions to support pregnant women and young families impacted by adversity exacerbated by the COVID-19 pandemic.

## Introduction

The COVID-19 pandemic has negatively impacted the health of parents and caregivers due to acute economic instability, social isolation, heightened health-related social needs, and increased responsibility over childcare and early childhood education in the setting of widespread child care and school disruptions and closures (1, 2). Due to a myriad of factors including systemic racism and socioeconomic inequities, these negative effects are disproportionately magnified for families from already marginalized communities. Black, Latinx, and Indigenous populations have experienced higher COVID-19 hospitalization and mortality rates than non-minority White populations (3–5). Additionally, since the passing of the Public Charge law in 2019, immigrant communities have shied away from healthcare and other essential social services in fear of deportation and other consequences, possibly exacerbating the negative health and social impacts of COVID-19 (6–8). Families who live below the federal poverty level experience increased financial instability from losing already low wage jobs, while others are required to continue working as essential workers in unsafe conditions (9–11). Oftentimes, these families also live in densely populated and highly polluted neighborhoods, increasing their risk of contracting COVID-19 (12, 13). Undeniably, COVID-19 exerts disparate impacts and exacerbates vulnerabilities in parents and families from already at-risk populations.

During these challenging times, families with young children, including pregnant women and those with children <6 years of age, are a vulnerable and understudied group. Literature has explored the stressors aggravated by COVID-19 on school age children and their families (1, 9, 10, 14). However, few studies have explored the adverse effects on perinatal maternal-infant health despite the unique vulnerabilities during this period of life having profound impact on longitudinal development and health outcomes. Increased stress and poor maternal mental health during pregnancy adversely alters the hypothalamic-pituitary-adrenocortical stress response system in the fetus (15). After birth, exposure to excessive adversity, sometimes called “toxic stress,” leads to disruption in neurocognitive development, metabolic dysregulation, and altered immune functions, all of which can lead to adverse chronic health outcomes in the absence of protective buffers (16–18). Sources of toxic stress can include but are not limited to abuse, neglect, and household dysfunction, all of which are magnified during the era of the COVID-19 pandemic (19–21). Fostering resilience is critical to mitigating the negative impacts of toxic stress, and protective buffers may include a supportive relationship with a committed caregiver, opportunities to build healthy coping skills, and strategies to reduce sources of toxic stress (22–26).

Although supportive family and community-based programs and COVID-19 resources exist, there is often a disconnect between the demonstrated need for support and connection to resources. Barriers such as the siloed domains of social support, mental health, fragmented communication during service referrals, and other structural inequities can reduce access to interventions. Furthermore, reduced in-person contact with clinical staff has hindered opportunities for resource navigation that normally occur during routine visits. Patient navigation is a patient-centered care delivery model first used in Harlem, NY to improve breast cancer screening and treatment outcomes in underserved populations (27). By removing barriers to care, patient navigation serves as a powerful strategy in mitigating health inequities and delivering quality care (27, 28). In the realm of women's healthcare, patient navigation programs have been successful in preventing maternal-child obesity, reducing preterm birth rates, and improving postpartum care delivery (29–31). We believe that patient navigation can be used to pave consolidated and integrative pathways to resources for pregnant women and families with children under age 6 during the COVID-19 pandemic.

Helping Us Grow Stronger (HUGS/Abrazos) is a community health center-based program using combined patient navigation and direct behavioral health support to address the social and behavioral health needs exacerbated by the COVID-19 pandemic among pregnant women and families with children under age 6. In this paper, we describe the design and implementation of HUGS/Abrazos, which aims to (1) provide pregnant women and young families who seek primary care at the Massachusetts General Hospital and affiliated community health care centers with resources and supports to help promote resilience and mitigate physical health, behavioral health, economic and other stressors that are likely to be exacerbated by the social isolation and economic challenges of the COVID-19 pandemic, and (2) build cross-systems linkages among community partners through the implementation of resource referral technology tools that facilitate closed loop communication between community-based organizations and providers and to track referrals.

## Context

**Figure 1:**
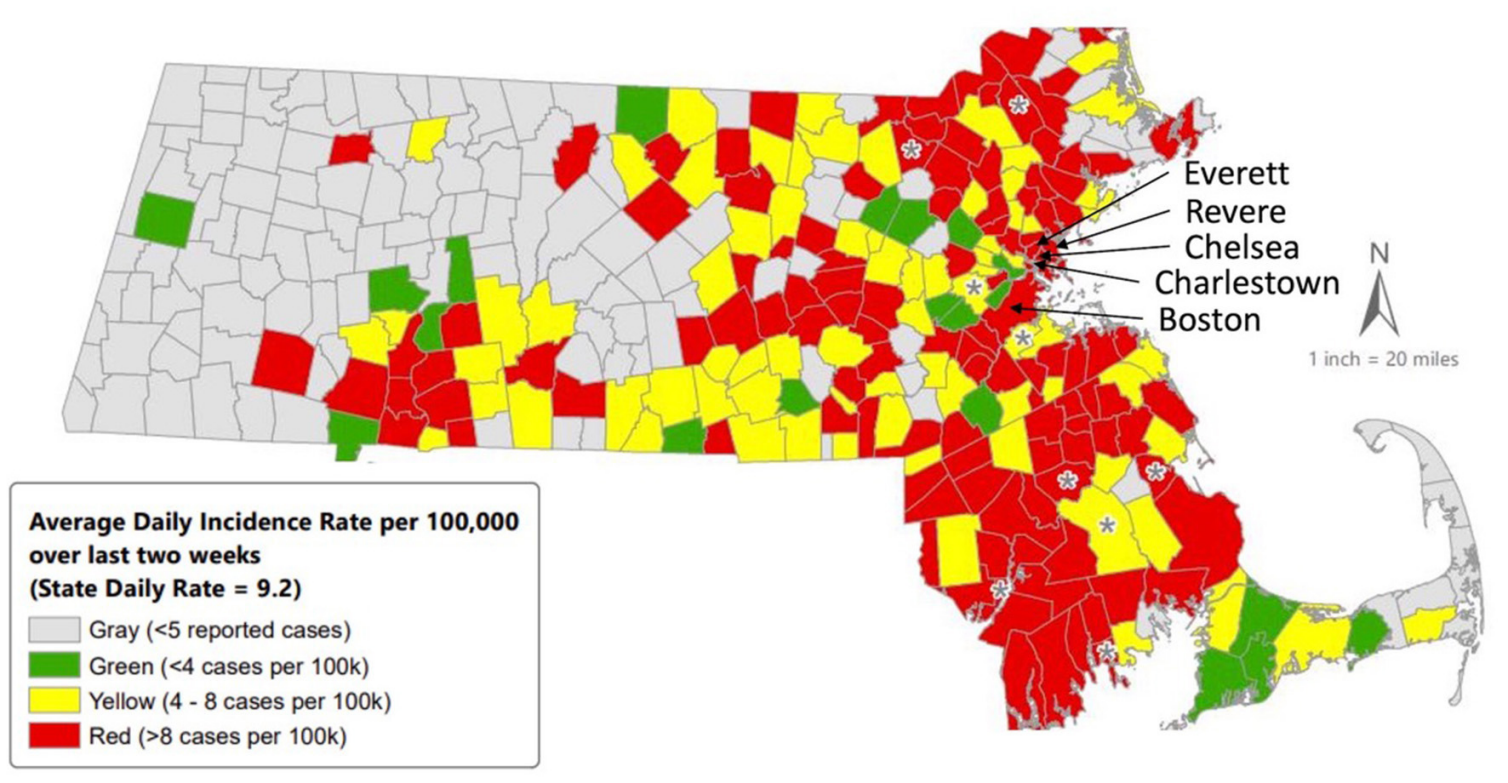
Average daily incidence rate (per 100,000, PCR only) for COVID-19 in Massachusetts by City/Town, 10/11/2020–10/24/2020. Citation: https://www.mass.gov/doc/weekly-covid-19-public-health-report-october-29-2020/download. Accessed November 17, 2020.

**Figure 2:**
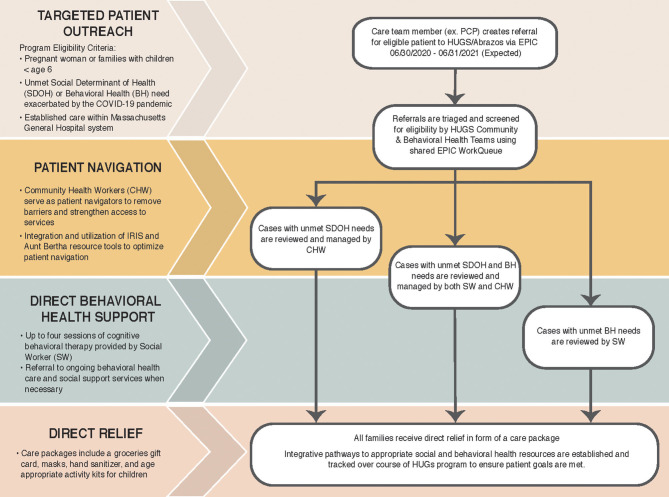
**Components of the HUGS/Abrazos COVID Emergency Response Program for Vulnerable Families**. Eligible patients and families are referred to the HUGS/Abrazos program by their primary care provider from a participating department-family medicine, pediatrics, OB/Gyn, or internal medicine-at the MGH Community Health Centers serving the towns of Chelsea, Revere, Everett, and Charlestown as well as the MGH Main Campus. CHW, Community Health Worker; BH, Behavioral Health; SW, Social Worker.

The primary objective of HUGS/Abrazos is to provide and connect pregnant women and families with children under age 6 with community resources and behavioral health support to alleviate stressors exacerbated by COVID-19. The program primarily serves the Massachusetts communities of Boston, Chelsea, Everett, Revere, and Charlestown, all of which have a higher than state average of families living in poverty, foreign born residents, and racial or ethnic minorities (Table 1). These communities have also been among the hardest hit by the COVID-19 pandemic in Massachusetts (Figure 1). Program eligibility criteria include pregnant women or families with children under age 6 that have demonstrated an unmet social or behavioral health need due to COVID-19 and have established care within the Massachusetts General Hospital system (Figure 2).

**Table 1:** Characteristics of community populations served by HUGS/Abrazos program.

	**MA**	**Chelsea**	**Revere**	**Everett**
**Race & Ethnicity**				
Total Population^a^	6,830,193	39,852	53,966	45,856
Non-Hispanic White^a^	72.2%	21.5%	54.5%	44.6%
Non-Hispanic Black^a^	6.8%	5.8%	5.2%	17.8%
Hispanic or LatinX (of any race)^a^	11.6%	66.9%	32.5%	26.5%
Foreign born^a^	16.5%	45.5%	38.7%	40.3%
Language other than English spoken at home^a^	23.6%	70.3%	51.1%	56.2%
**Income/Economics**				
Median Family Income^a^	$101,548	$57,216	$68,187	$64,819
Median Family Income with own children (<18Y)^a^	$104,496	$48,391	$60,072	$49,066
% Families living below federal poverty level^a^	7.5%	15.5%	9.4%	12.3%
% Families with children <5 years old living below federal poverty level^a^	10.4%	14.4%	11.9%	42.1%
% Families receiving public assistance^a^	11.0%	24.9%	14.4%	20.4%
% Children living in households with public assistance^a^	20.7%	33.9%	28.3%	31.0%
**COVID-19 Statistics**				
Total Positive Cases^b^	150,498	3,939	3,201	2,636
Percent Positivity^b^	1.55%	4.31%	6.15%	4.45%
Average Daily Incidence Rate per 100,000^b^	11.8	32.6	39.4	24.4

*aBased on American Community Survey 2014-2018 5-Year Estimates*.

*bBased on Massachusetts Department of Public Health Weekly COVID-19 Public Health Report – October 29, 2020. Percent positivity and average daily incidence rate is reported based on a 14-day average. https://www.mass.gov/doc/weekly-covid-19-public-health-report-october-29-2020/download. Accessed November 19, 2020*.

Program implementation required stakeholder engagement, adequate staffing, and overcoming the unique challenges of patient navigation during the COVID-19 pandemic, including providing services in a virtual environment. We engaged providers and staff across multiple departments–OB/Gyn, Pediatrics, Community Health Improvement, Population Health, the Healthy Chelsea community coalition, and Behavioral Health–as stakeholders and collaborators in the HUGS/Abrazos program. Stakeholders met biweekly prior to the launch of the program to develop program eligibility criteria, goals, and workflow. From the onset of program development, we realized the importance of adequate staffing to ensure successful patient navigation and behavioral health support amidst a pandemic hospital surge. We hired a social worker to address behavioral health needs, and engaged community health workers from within the MGH Center for Community Health Improvement to serve as HUGS patient navigators. By integrating preexisting staff and leveraging their multilingual capabilities, familiarity with the hospital systems, and experience in working with our targeted patient populations, we achieved rapid program implementation, cross-departmental communication, and increased chances of sustainability in the future. Community health workers are required to use skills such as problem solving, tracking, and collaboration to appropriately and effectively connect patients with necessary resources.

Unlike previous navigation programs at MGH, we implemented two resource tools aiming to facilitate patient navigation: Aunt Bertha, a shared resource portal, and Integrated Referral Information System (IRIS), a web-based direct referral platform (32, 33). These technological tools facilitated better knowledge and staff resource sharing within MGH and increased patient connection to resources via direct referral. Patient navigators compile relevant resources in shared folders within Aunt Bertha for the staffing team to see, and IRIS allowed community health workers to directly refer patients to the select community-based resources patients need. Use of IRIS promotes increasing understanding between community health workers and patients regarding referral status, eligibility, availability, and wait time for services. Training for both tools occurred through asynchronous and synchronous learning opportunities, as well as sharing of best practices.

## Programmatic Elements

### Targeted Patient Outreach

Initial referrals to the HUGS/Abrazos program are made through the electronic health record by care team members (e.g., primary care doctors) in the pediatrics, OB/Gyn, internal medicine, family medicine, and other departments. Prior to its launch, the HUGS/Abrazos program goals, benefits, and eligibility requirements were shared with providers through department-wide meetings, email announcements and newsletters. Eligibility requirements for the program include: (1) patient is pregnant or the parent of a child under 6 years of age, (2) parent or child has a primary care or obstetric provider established at MGH, and 3) the parent has a history of depression/anxiety or the parent-child dyad has a social health need (e.g., food, infant supplies such as diapers, utilities).

A referral order was developed and integrated into the Epic electronic health record system allowing patients to be referred for behavioral health, social determinant of health needs, or both. Incoming referrals are triaged by community health and behavioral health supervisors for assignment to the HUGS community health workers and social worker. Using a team-based approach, cases are assigned to a community health worker when the specified needs are related to unmet social needs (i.e., food insecurity) and/or a social worker when the needs are related to unmet behavioral health needs (i.e., anxiety, depression). Cases may be initially reviewed by a community health worker to address social needs, and subsequently referred to the social worker if behavioral health needs are elucidated in later encounters and vice versa.

The number of encounters each patient has with the HUGS/Abrazos team varies depending on the reason for referral. In general, the program was designed to provide effective, short-term crisis management support with up to three touchpoints with the community health team and four with the behavioral health team. In order to ensure effective tracking of referrals and transparent communication among all care providers, we developed a program-specific form within the Electronic Medical Record. The form serves as a centralized document that stores all clinical and evaluation notes from the community health workers and/or social worker, and can be accessed by the patient's medical care team to facilitate communication among all care team members serving the patient. Lastly, it allows for easy transfer of patient information from their medical chart to each note for documentation purposes.

### Patient Navigation by Community Health Workers

Community health workers engage patients in up to three separate telephone encounters to identify and address the most urgent social determinant of health needs to provide short-term connection to resources. During the initial session, community health workers assess for unmet social needs through a 10-item questionnaire previously utilized by COVID-19 respiratory illness clinic staff (Table 2). To assess for other imminent concerns, patients are screened for interpersonal violence, substance use disorder, and depression. Interpersonal violence is assessed using a 2-item screener and substance use disorder is assessed using a 3-item screener. If the patient screens positive for either, the referring provider is immediately notified in a confidential manner. Depression is screened using a PHQ-2, and if positive, the patient is offered the opportunity to connect with the social worker for behavioral health support. Following the initial encounter, community health workers utilize resource referral tools to connect patients with community resources to support their needs.

**Table 2:** 10-item screening domains for social determinants of health related to COVID-19.

**Domain of health-related social needs**	**Questions used by community health workers to assess needs**
Employment	Are you currently working? Is it full-time?
Food insecurity	Are you worried about food?
Rent or utility assistance	Do you have any worries about your home? How are you doing with rent? Are you worried about not having enough money to pay next month's rent? Are you worried about electricity/heating?
Medication access	Are you worried about medications?
Hygiene	Are you worried about soap, clean water, hand sanitizer?
Baby supplies	Are you worried about baby supplies (i.e., formula, diapers, wipes)?
Transportation to health care	How do you get to your doctor's appointments?
Social support	Who lives with you at home? Who do you to talk to or turn to for support?
Child care	Who can help you when you need someone to care for your child?
Additional concerns	Do you have other concerns you would like to discuss?

In the second and third encounters, the community health worker follows up with the patient regarding resource referrals. At the third touchpoint, the community health worker coaches the caregiver on fostering their child's early development by asking about the parent-child relationship, identifying any developmental concerns, and discussing strategies to promote early development such as reading to their child. Parents are also provided resources about early development such as the Text4Baby or The Boston Basics text messaging campaigns, and the CDC milestone tracker app (34–36). In this final session, the patient and community health worker also review progress made and identify any outstanding unmet needs. If necessary, the patient is connected to long term support by notifying the provider and other care team members. Between scheduled encounters, the community health worker may also communicate with the patient via text messaging to exchange information on resources, address brief questions, and confirm appointments.

Community health workers are using two new technological tools during the patient navigation process that improve knowledge, enhance staff resource sharing, and increase connection to resources via direct referral. Aunt Bertha is a web-based resource portal which is free to all, though a custom account has been created for MGH staff to allow for an integrated workflow. Community health workers use Aunt Bertha to search for desired community resources (i.e., food bank), which they then save to early childhood shared folders to reduce redundant searching across staff, organize resource experience comments, and quickly find past-utilized agencies by geographic domain. CHWs can text and email resources directly to families via Aunt Bertha, which offers an organized space for staff to keep track of and follow-up on soft referrals. Patients can also then use Aunt Bertha to search for resources themselves, which has the potential to make them more self-sufficient over time. When a CHW identifies a relevant resource to a community-based organization (CBO) (e.g., Head Start), they can use IRIS to directly refer patients to that agency, thereby ensuring that the connection will be made. The 15 agencies in IRIS have committed to help families connect to needed services by increasing understanding regarding referral status, eligibility, availability, and wait time for services. Once a referral is placed by a CHW in IRIS, a representative from the referred agency immediately receives the referral through the HIPAA-compliant system. The referred agency responds to the referral request typically in 5 business days, after which a representative from the agency will reach out to the patient and the referral status on IRIS is updated to inform the referral source whether the patient has accessed the recommended service. While patients do not access the IRIS system directly, the eligibility, availability and wait time information in the IRIS organization profiles are shared with families to ensure understanding of the scope of services and next steps.

### Direct Behavioral Health Support by a Social Worker

HUGS/Abrazos also provides immediate and direct behavioral health support by a master's level social worker (Figure 2). The HUGS social worker is a behavioral health provider whose role is to ameliorate patients' behavioral health needs. When eligible patients with behavioral health disorders such as depression and/or anxiety are referred to the HUGS program, the social worker can provide up to four cognitive behavioral therapy sessions to assess and address underlying psychosocial challenges. The social worker also asks patients' how COVID-19 has affected energy and stress levels, how it has changed their routines and their child's routines related to eating, sleeping, screen time, and play and outdoor time, and what resources could be helpful to the family during the pandemic. In the course of this therapeutic alliance, the social worker can provide crisis management through assessment of safety, domestic violence, and child abuse concerns, followed by appropriate psychoeducation and support programming for the identified crisis. The cognitive behavioral therapy sessions serve as an immediate strategy to alleviate behavioral health stressors and foster necessary coping mechanisms to build resilience within families.

When indicated, the HUGS/Abrazos social worker may refer patients for long term behavioral health support, or to services such as substance use treatment programs, legal support, and financial services. In addition, the social worker may connect the patient with the community health worker if there are unmet social needs impacting the patient's progress. In such cases, the social worker and community health worker act as team and communicate progress and plans for the patient. The social worker and community health workers also meet monthly to discuss collaborative cases and ensure the seamless support of patients.

### Direct Relief and Care Packages

**Figure 3:**
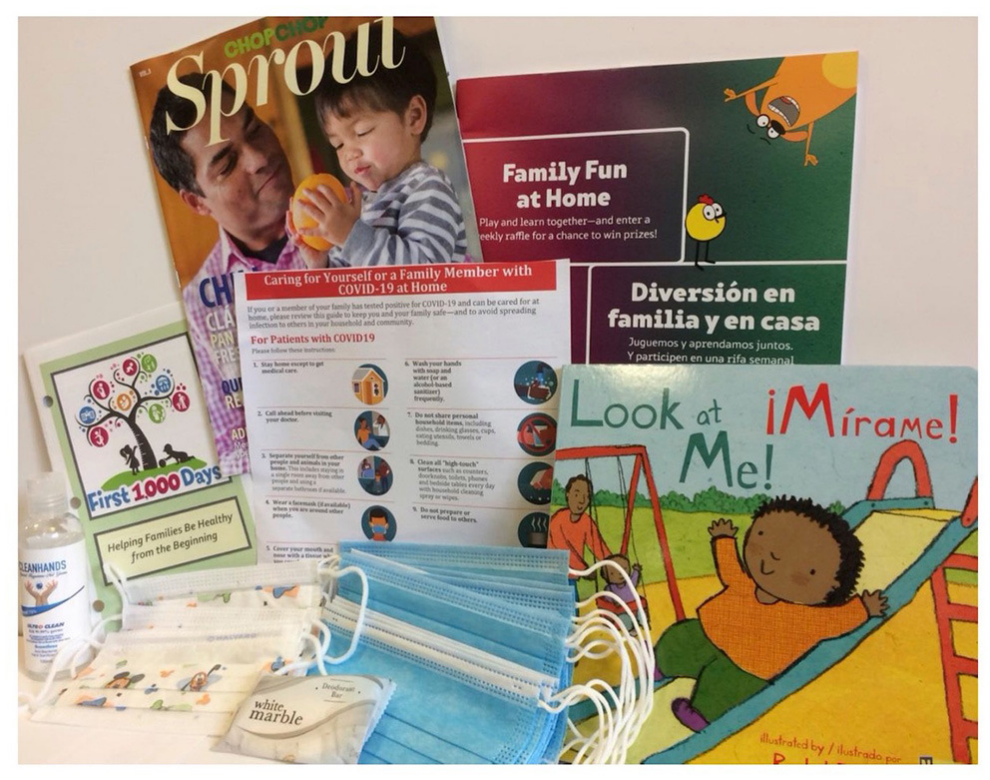
HUGS/Abrazos COVID-19 care package for families.

All families engaged in the HUGS/Abrazos program receive a care package including (1) a $50 grocery gift card, (2) face masks for children and adults, (3) hand sanitizer, (4) a handout on COVID-19 care for family members, (5) a bi-lingual children's book, (6) a ChopChop healthy cooking magazine, (7) First 1,000 Days educational booklets to support healthy pregnancy and infancy (Figure 3). All materials are available to patients in English and Spanish. The care packages provided immediate food assistance given the high prevalence of food insecurity, and encouraged healthy practices during the COVID-19 pandemic.

## Discussion

Ample evidence supports the importance of fostering resilience in the face of early life adversity as a means to ameliorate the negative impacts of toxic stress (22–26). There has never been more urgency than now in providing the necessary tools and interventions to support families impacted by toxic stress exacerbated by the COVID-19 pandemic. The HUGS/Abrazos program utilizes a combination of targeted patient navigation and immediate behavioral health support to improve the most pressing social and behavioral health needs for vulnerable populations, while simultaneously building pathways toward long term support.

### Program Innovation

The HUGS/Abrazos program is innovative in several ways. First, our focus on serving pregnant women and young children is particularly important since this population has been thus far understudied during the era of the COVID-19 pandemic, despite the profound implications on chronic health outcomes. Second, from development and throughout implementation, we elucidated input from all stakeholders–primary care, behavioral health, and community health–to understand the most pressing needs of the community and to address the specific barriers we needed to overcome. Leveraging interprofessional expertise, preexisting resources, and support systems relating to early childhood health informed our workflow framework in such a way that allowed us to progress rapidly from conception to actual implementation of the program. Third, we adopted two innovative resource tools–Aunt Bertha and IRIS–to overcome the siloed nature of community resources, form close-looped communication amongst involved parties, and track referrals and patient goals to ensure delivery of quality care. With the COVID-19 pandemic causing termination of some existing services while catalyzing implementation of new emergency resources, it was difficult for patients and clinicians alike to keep track of what resources are currently available. The usage of the Aunt Bertha and IRIS systems were integral in overcoming these challenges. Lastly, the HUGS/Abrazos programs is one of the first within the hospital system to accept patients for resource navigation across all community health centers, catalyzing other programs to do the same.

Utilization of multidisciplinary teams, resourceful repurposing of existing support staff, as well as innovative technological tools that provide up-to-date listings and availability of community resources were the cornerstones driving the efficient implementation of HUGS/Abrazos. These basic principles used for HUGS/Abrazos implementation can be applied to any program seeking to provide rapid response relief to populations suffering from public health crises. Furthermore, the multidisciplinary structure of the HUGS/Abrazos program and the utilization of Aunt Bertha and IRIS can extend beyond the acute phase of the pandemic to provide sustained support for vulnerable young families as the stressors of the pandemic will likely continue beyond its acute phase.

### Conceptual and Methodological Constraints

Though the HUGS/Abrazos program possesses several strengths and innovations, there are limitations to note. The recruitment of existing community health workers has offered advantages such as leveraging the team's multilingual capabilities, familiarity of the system, and a rapid start to the program, but staff also face competing demands as they support other community health initiatives. This represented a key barrier in program staffing that was resolved through ensuring the buy-in from clinical supervisors, who then encouraged staff supporting HUGS/Abrazos. In the future, the sustainability of HUGS/Abrazos program staffing will remain contingent upon continued support from key supervising roles.

Another key barrier in program implementation was sufficient patient participation amidst competing parental responsibilities at home, such as increased at home childcare and remote schooling during the COVID-19 pandemic. This was resolved through offering evening behavioral health appointments, conducting “mini visits” to deliver the most salient components of the program, and offering video modules for practicing mindfulness at the patient's convenience. Nonetheless, all patient outreach has occurred via video or phone, potentially posing a barrier to services for patients that have limited access to technology. Similarly, due to the nature of remote work, direct relief in the form of gift cards and care packages have been mailed, rather than provided on-site to patients, and thus delaying delivery. Most notably, while HUGS/Abrazos lays the groundwork for multidisciplinary crisis mitigation in pregnancy and early childhood, complete scale-up and sustainability of the program is dependent on sustained funding to support staff providing these services. To demonstrate the value of HUGS/Abrazos and advocate for sustainability, we are evaluating the program as described below.

### Evaluation

We will evaluate the HUGS/Abrazos Program using a mixed-methods approach, guided by the Reach, Effectiveness, Adoption, Implementation, and Maintenance (RE-AIM) framework (37). The RE-AIM framework recognizes the complexity of real-world settings in evaluation, and leverages the combined evaluation of each domain to appropriately evaluate public health promotion programs. For our program, the domains of RE-AIM will be assessed through electronic health record documentation, interviews and surveys with program staff, and administrative records. This project was undertaken as a Quality Improvement Initiative at Massachusetts General Hospital, and as such was not formally supervised by the Institutional Review Board per their policies.

*Reach* will be evaluated as the number of program referrals and demographic characterization of the population served. *Effectiveness* will be evaluated based on number of completed interactions with program staff, referrals made and resources provided. *Adoption* will be evaluated on the number of providers and departments making program referrals and usage of the newly implemented resource referral tools, Aunt Bertha and IRIS. *Implementation* will look at fidelity, acceptability and appropriateness of the program. *Maintenance* will assess the program's ability to be sustained after the initial funding period ends, including exploration of the ability for community health worker and/or social worker support to be medically billed.

## Conclusions

Through the application of patient navigation supports and direct behavioral health services, the HUGS/Abrazos program seeks to fill an important void of providing the necessary tools and interventions to support pregnant women and young families impacted by toxic stress exacerbated by the COVID-19 pandemic. The program is likely to serve as a catalyst for other social and behavioral support programs based in the clinical care setting targeting vulnerable young families.

## Data Availability Statement

The original contributions generated for this study are included in the article/Supplementary Material, further inquiries can be directed to the corresponding author/s.

## Author Contributions

EMT, CT, GMK, AAB, MLH, MEP, MYP, and AI: each author made substantial contributions to the conception or design of the work. ML, FN, MEP, SNP, WL, CT, and EMT: drafting the manuscript. All authors revising and giving approval of the final manuscript.

## Conflict of Interest

The authors declare that the research was conducted in the absence of any commercial or financial relationships that could be construed as a potential conflict of interest.
